# Effect of Gelatin Source on Hydrogel Properties and Cell Viability: A Comparison of Bovine, Porcine, and Cold-Water Fish Gelatin Hydrogels

**DOI:** 10.3390/gels12090789

**Published:** 2026-09-01

**Authors:** Antonio David Abreu-Rejón, Abril Alexa Olivares-Ramírez, Wilberth Antonio Herrera-Kao, Nayeli Rodríguez-Fuentes, José Manuel Cervantes-Uc, Rogelio Rodríguez-Rodríguez

**Affiliations:** 1 SECIHTI-Centro de Investigación Científica de Yucatán, A.C, Unidad de Materiales, Calle 43 No. 130, Col. Chuburná de Hidalgo, 97205 Merida, Yucatán, Mexico; nayeli.rodriguez@cicy.mx; 2 Centro de Investigación Científica de Yucatán, A.C, Unidad de Materiales, Calle 43 No. 130, Col. Chuburná de Hidalgo, 97205 Merida, Yucatán, Mexicogywahkao@cicy.mx (W.A.H.-K.); 3 Departamento de Ciencias Naturales y Exactas, Universidad de Guadalajara, Carretera Guadalajara-Ameca Km 45.5, 46600 Ameca, Jalisco, Mexico

**Keywords:** gelatin, bovine, porcine, fish, osteoblasts, fibroblasts, viability, adhesion

## Abstract

The selection of a gelatin source is a critical decision in biomaterial design, as its origin dictates key physicochemical and biological properties. This study systematically compares gelatin hydrogels from bovine, porcine, and cold-water fish sources to elucidate how biological origin influences performance as biomaterials. Comprehensive characterization confirmed that the distinct amino acid profiles of each source, verified by FTIR and Raman spectroscopy, govern their material behavior. Fish gelatin exhibited a lower primary amine content (approximately 70% of the free primary amine content of bovine gelatin, quantified by ninhydrin assay), reduced thermal stability (TGA), and a less ordered structural hierarchy (XRD) compared to mammalian gelatins. In cell culture, fibroblast viability was similar across all hydrogels, whereas osteoblast viability was lower on fish gelatin, and both cell types exhibited a more contracted morphology on fish gelatin hydrogels. In pull-off tests, fish gelatin hydrogels displayed a lower slope (−0.32 ± 0.03 N/mm) and a more gradual force decay, remaining attached to the probe over a larger displacement than the mammalian hydrogels (−1.73 ± 0.23 N/mm for porcine and −0.99 ± 0.40 N/mm for bovine). These observations suggest that, although fish gelatin remains a promising alternative to mammalian sources for certain applications, its structural and biological limitations require further material optimization. Notably, the pull-off behavior further suggests a possible niche as a bio-adhesive.

## 1. Introduction

Gelatin hydrogels are widely used in biomedical applications because of their inherent biocompatibility, biodegradability, and low immunogenicity [1]. Gelatin is derived from the partial hydrolysis of collagen, the most abundant protein in animal tissues. Consequently, this biopolymer can be sourced from various animals, with bovine, porcine, and fish being the most prevalent [2,3]. Gelatin is categorized into two types based on the pre-treatment of collagen: type A and type B. Type A gelatin, produced by acid treatment, has an isoelectric point between pH 6 and 9 and is typically derived from the less cross-linked collagen of pig skin. In contrast, type B gelatin is produced through alkaline treatment, has an isoelectric point around pH 5, and is suitable for processing the more complex collagen structure of bovine hides [4]. Chemically, gelatin is composed of 18 different amino acids, with glycine, proline, and hydroxyproline constituting the majority of its composition [5].

Although all gelatins share a similar triple-helical foundation, their amino acid composition varies significantly with the source and extraction method, in particular the content of imino acids proline and hydroxyproline. These differences impart distinct physicochemical properties, including molecular weight distribution, thermal stability, and gelation behavior [4,6,7]. For instance, mammalian gelatins (bovine and porcine) typically exhibit higher gel strength and thermal stability compared to fish gelatin, which has a lower melting point and gel strength due to its reduced imino acid content [8,9,10]. Such variations are critical because they define the stiffness, porosity, and overall physical microenvironment that cells experience within a hydrogel scaffold.

In biomedical applications, the cellular response to a hydrogel scaffold is fundamental. Gelatin matrices influence cells through integrated chemical (e.g., bioactive ligand density) and physical cues (e.g., stiffness, porosity). Differences in gelatin sources may lead to varying cellular responses, as each source presents distinct structural features, amino acid residues, and degradation rates [9,11,12]. Previous studies have shown variable cell response depending on the gelatin source, suggesting that a comparative analysis of their effects on cell viability could provide essential insights for optimizing gelatin-based hydrogels for tissue engineering [13,14,15]. For instance, Aljaber et al. [16] synthesized gelatin methacryloyl (GelMA) hydrogels from porcine, bovine, and fish sources. They determined that the bovine-derived GelMA exhibited superior mechanical properties. While porcine and bovine gelatins induced slightly higher cell viability at 24 and 96 h compared to fish gelatin, the difference was not statistically significant. In a study by Wang et al. [17], porcine-derived GelMA hydrogels supported a higher proliferation rate of NIH 3T3 fibroblasts after 72 h compared to those derived from fish gelatin. Furthermore, Pahoff et al. [14] developed porcine and bovine GelMA/hyaluronic acid hydrogels and found that the bovine-derived version most closely mimicked the properties of native articular cartilage after 28 days in culture.

In this study, we undertake a comprehensive physicochemical characterization of gelatin hydrogels from bovine, porcine, and cold-water fish sources and their effects on cell viability with the aim of providing insights into how the different chemical and physical properties of these gelatins influence cellular response and contribute to the development of more effective gelatin-based biomaterials with optimized properties for use in regenerative medicine.

## 2. Results and Discussion

**Figure 1:**
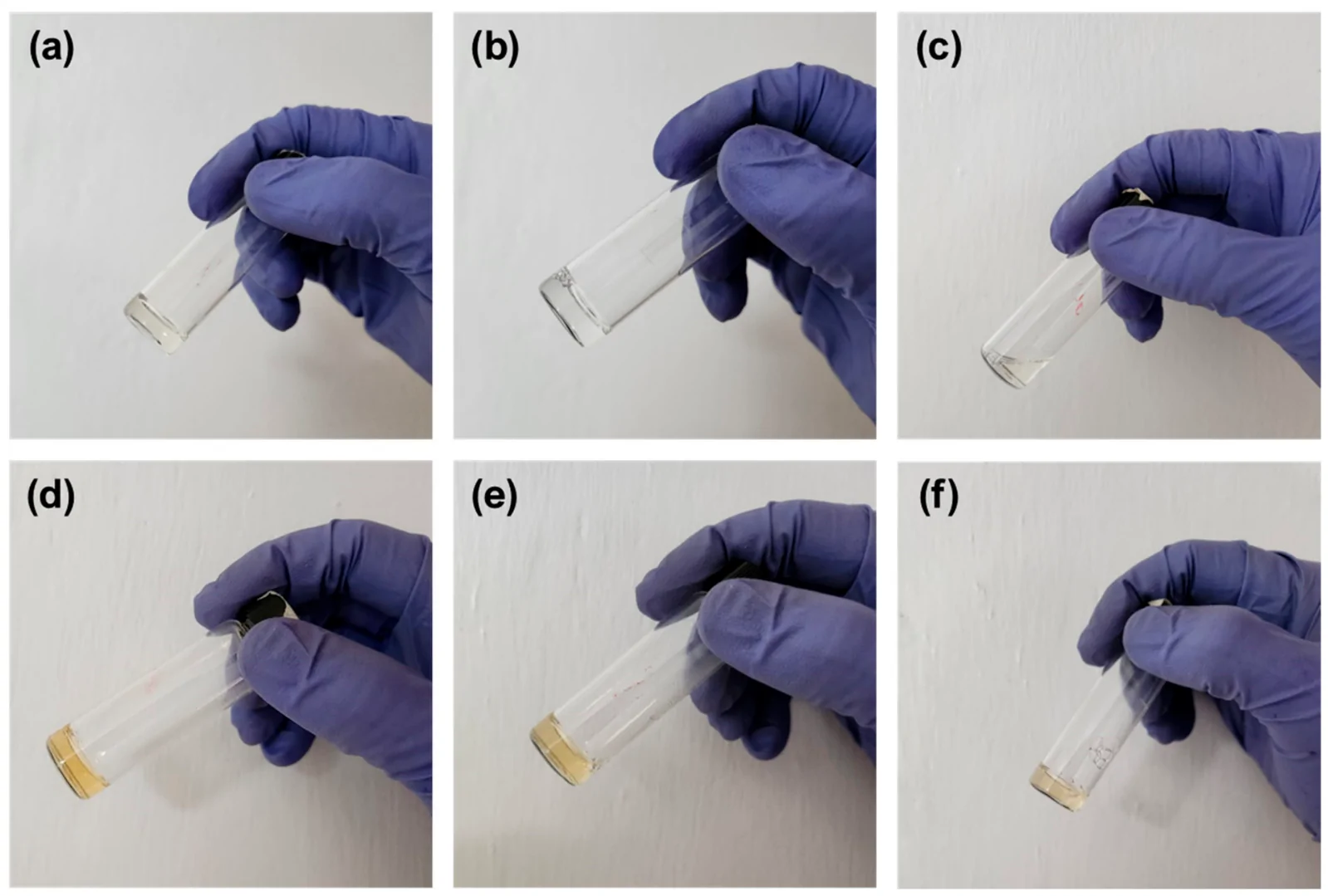
Macroscopic appearance of gelatin hydrogels prepared at 25 °C. Panels (**a**–**c**) show uncrosslinked gelatin samples: (**a**) bovine and (**b**) porcine gelatin formed transparent, self-supporting hydrogels, whereas (**c**) cold-water fish gelatin failed to gel and remained liquid under these conditions, consistent with its lower imino acid content. Panels (**d**–**f**) show GTA-crosslinked gelatin hydrogels: (**d**) bovine, (**e**) porcine, and (**f**) cold-water fish gelatin hydrogels exhibited a yellowish coloration characteristic of imine (Schiff base) bond formation.

Representative photographs of the gelatin hydrogels are presented in Figure 1. Visually, uncrosslinked hydrogels are transparent, while their crosslinked counterparts exhibit a distinct yellow hue, a characteristic indicative of imine bond (Schiff base) formation. Furthermore, the mammalian gelatins form stable hydrogels at room temperature (25 °C), a property conferred by their high imino acid (proline and hydroxyproline) content (Figure 1a,b). In contrast, cold-water fish gelatin, with its inherently lower imino acid content, fails to gel under these conditions (Figure 1c). When crosslinked with the same glutaraldehyde (GTA) concentration (2% *w*/*w*) employed for mammalian gelatins, fish gelatin hydrogels initially formed but were not stable under physiological conditions; they dissolved when incubated at 37 °C for 24 h after cell seeding. Consequently, a twofold increase in GTA concentration (4% *w*/*w*) was required to produce fish gelatin hydrogels that remained stable for cell culture.

### 2.1. Free Primary Amine Groups and Crosslinking Degree

**Figure 2:**
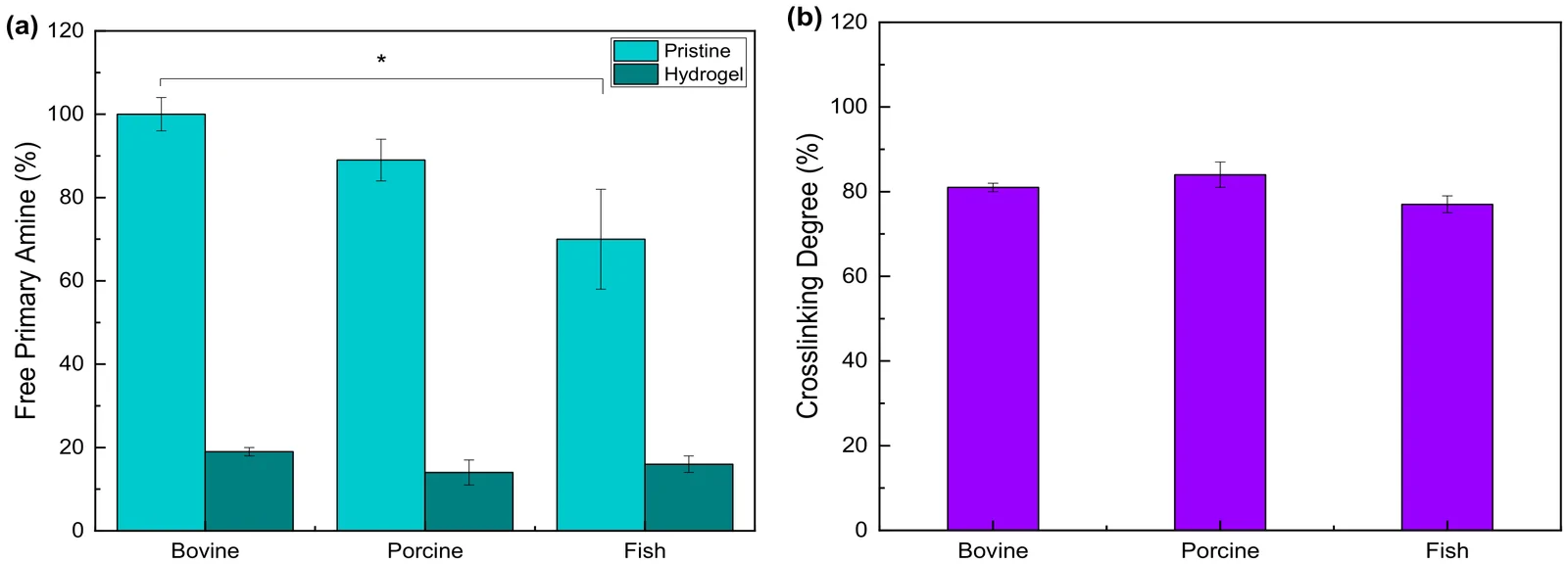
Quantification of free primary amines of gelatins through ninhydrin test (**a**) and crosslinking degree of hydrogels (**b**). (*): statistical difference, *p* < 0.05.

**Figure 3:**
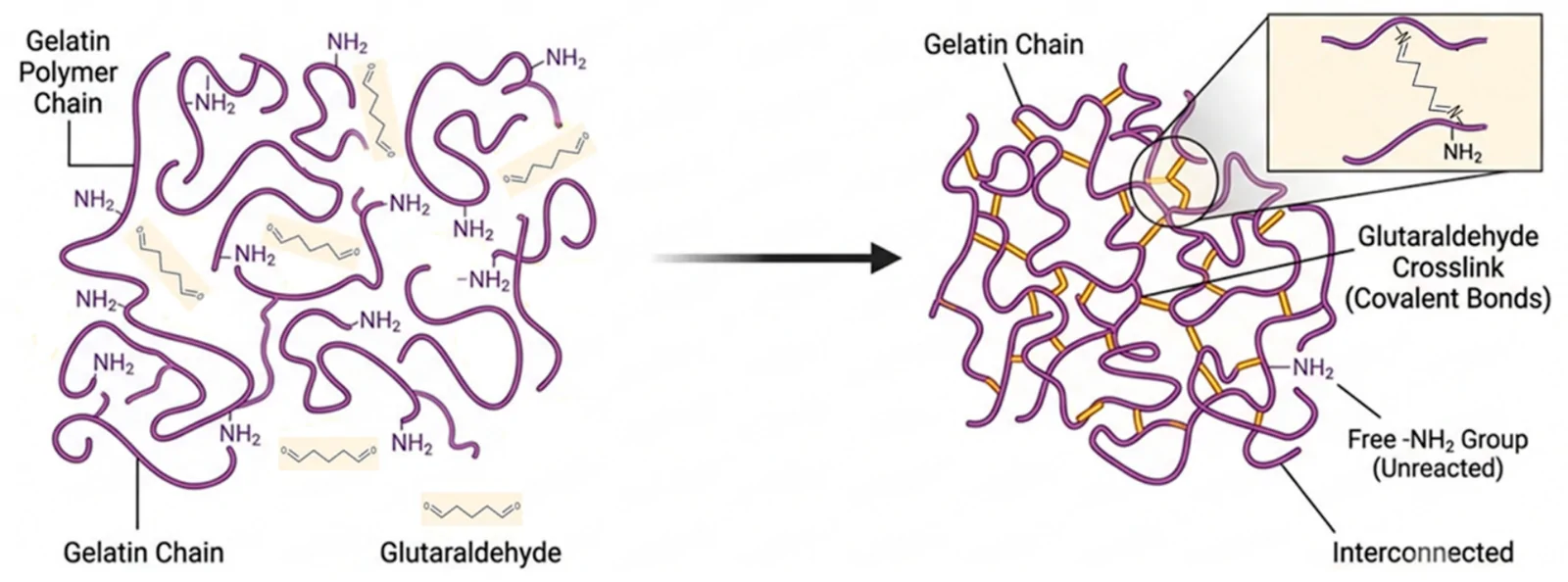
Schematic representation of the crosslinking reaction between gelatin chains and GTA. The left panel depicts the initial state, in which gelatin chains and GTA molecules are dispersed homogeneously. The right panel shows the resulting crosslinked network, in which GTA molecules bridge gelatin chains by reacting aldehyde groups with primary amine groups to form imine (Schiff base) linkages. For clarity, only representative reactive sites are shown.

The results of the ninhydrin assay for the gelatins are presented in Figure 2. Bovine gelatin exhibited the highest content of free primary amines, followed by porcine gelatin with approximately 89% of the amount measured in bovine gelatin, whereas fish gelatin displayed a significantly lower value, corresponding to 70% of the bovine gelatin content. This finding is in agreement with the work of Serafim et al. [18], who reported primary amine contents of 6.5 × 10^−4^ moles NH_2_ per gram of protein for bovine gelatin and 4.1 × 10^−4^ moles NH_2_ per gram of protein for fish gelatin. The observed variation in primary amine content can be attributed to differences in the amino acid profiles of the gelatins. This parameter is essential because the availability of primary amine groups directly governs cross-linking efficiency during hydrogel formation. The content of free primary amines decreased in hydrogels due to the reaction of aldehyde groups with primary amines, resulting in the formation of imine linkages (see Figure 3). The degree of crosslinking, defined here as the percentage of available primary amines consumed, was comparable among all hydrogel formulations (Figure 2b), although fish gelatin hydrogels exhibited a slightly lower crosslinking degree. This observation is noteworthy because the amount of GTA used to crosslink the fish gelatin was twice that used for the mammalian gelatins, a difference that can be ascribed to the lower initial number of free primary amines in fish gelatin. Nevertheless, the stabilization of fish gelatin hydrogels at the higher GTA concentration may not rely exclusively on amine crosslinking. The increased GTA concentration could promote glutaraldehyde self-polymerization and aldol condensation, leading to oligomeric species that become physically entangled or form additional, non-amine linkages within the network [19,20]. These oligomerization pathways can also increase the probability of effective amine crosslinking despite the lower density of primary amines. Because the ninhydrin assay does not capture these contributions, they may help explain the observed gel stability and swelling behavior of fish gelatin hydrogels without implying a more densely amine-crosslinked network.

### 2.2. FTIR

**Figure 4:**
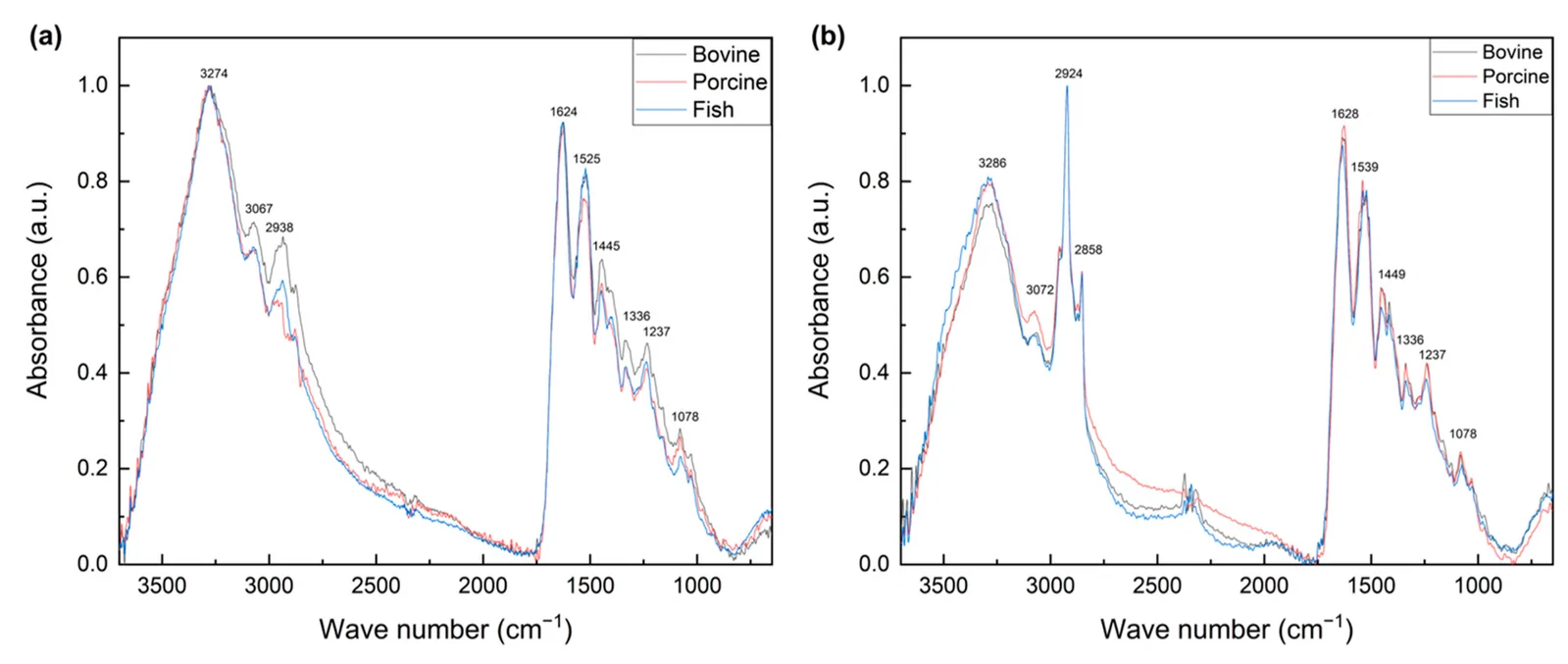
Fourier transform infrared (FTIR) spectra of gelatin samples. (**a**) Spectra of pristine, uncrosslinked gelatins from bovine, porcine, and cold-water fish sources, showing characteristic amide bands associated with the polypeptide backbone. (**b**) Spectra of the corresponding GTA-crosslinked gelatin hydrogels. Changes in band intensities and positions following crosslinking, particularly in the amide A, amide B, and C–H stretching regions, reflect the consumption of free primary amines and the incorporation of GTA into the polymer network. All spectra were normalized to their maximum absorbance for comparison.

FTIR spectra of gelatins are shown in Figure 4. The characteristic protein bands can be observed at 3274 and 3067 cm^−1^, corresponding to N-H stretching (amide A and B); 2938 and 2876 cm^−1^ attributed to C-H stretching; 1624 cm^−1^ due to C=O stretching (amide I); 1525 cm^−1^ corresponding to N-H bending (amide II); 1445 cm^−1^ due to C-H bending; 1336 cm^−1^ attributed to O-H bending; 1237 cm^−1^ corresponding to C-N stretching (amide III); and 1078 cm^−1^ due to C-O stretching. The FTIR spectra of the pristine gelatins (Figure 4a) exhibited variations in band intensity across the different gelatin sources. Bovine gelatin generally exhibited higher absorbance at 3067, 2938, 1445, 1336, 1237, and 1078 cm^−1^. Fish gelatin showed more intense bands at 2938 and 1237 cm^−1^ than porcine gelatin. Conversely, porcine gelatin had a higher absorbance at 1078 cm^−1^ than fish gelatin but the lowest intensity of all at 1525 cm^−1^. These spectral differences originate from the distinct amino acid compositions of the gelatins. The alkaline process used to produce Type B gelatin (bovine) promotes the deamidation of asparagine and glutamine residues, converting their amide groups into carboxyl groups and thereby increasing the content of aspartate and glutamate [1,4]. Furthermore, fish gelatin has a lower proportion of proline and hydroxyproline but a higher content of serine and glycine than mammalian gelatins [2,6]. Collectively, these variations in functional groups and side chains account for the differences in intensity observed in the infrared absorption bands.

Following the preparation of GTA-crosslinked hydrogels, marked changes are observed in the FTIR spectra of the gelatins, as illustrated in Figure 4b. The intensities of the amide A and amide B bands diminish, whereas the C–H stretching bands at 2938 and 2876 cm^−1^ increase in intensity and shift to lower wavenumbers (2924 and 2858 cm^−1^, respectively). These spectral alterations arise primarily from crosslinking with GTA: the aldehyde groups of GTA react with the primary amines of gelatin to form imine linkages, thereby reducing the number of N–H groups. Simultaneously, incorporating GTA molecules into the hydrogel network increases the number of C–H bonds.

### 2.3. Raman Spectroscopy

**Figure 5:**
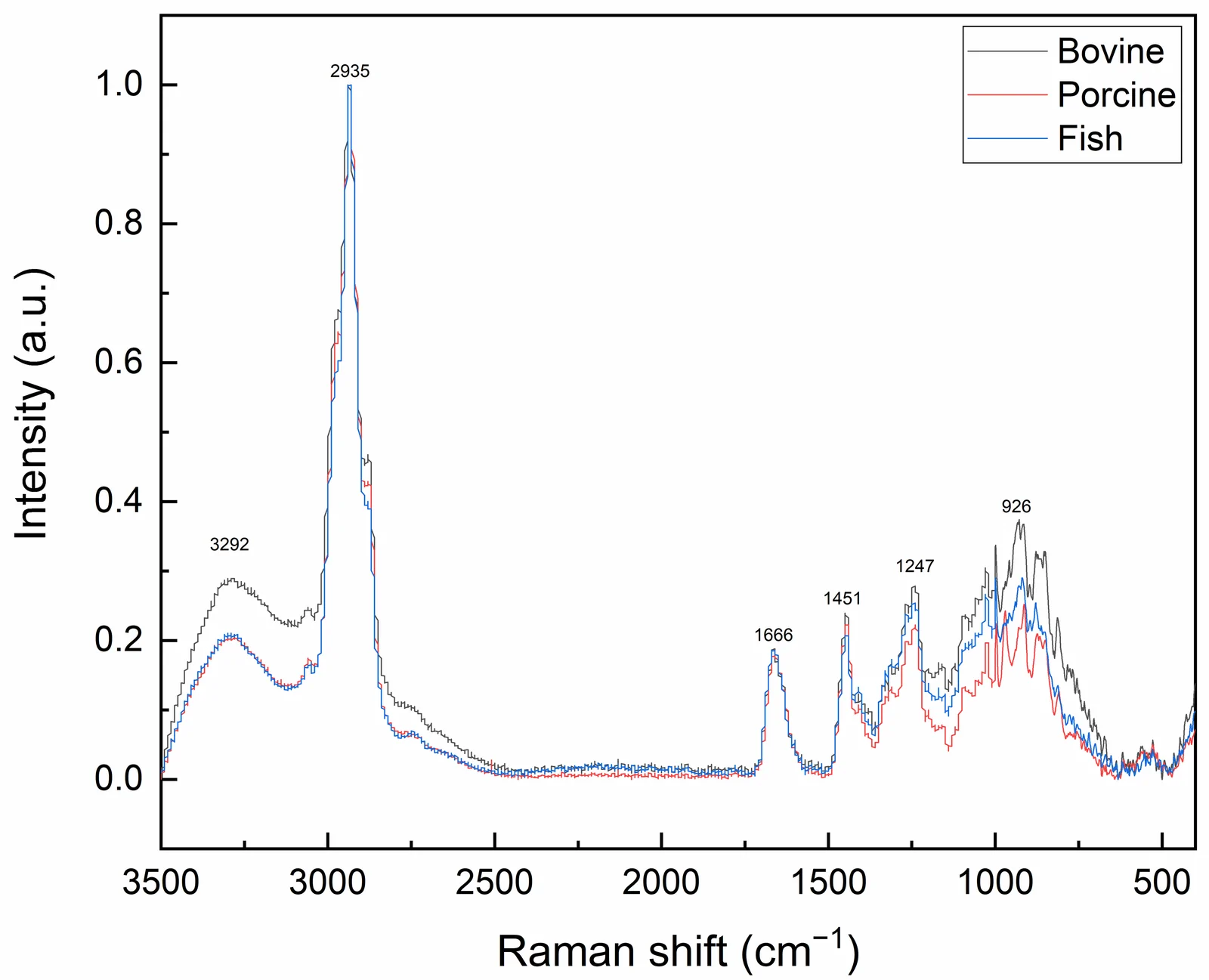
Raman spectra of pristine gelatins from bovine, porcine, and cold-water fish sources. The spectra display characteristic vibrational bands associated with the polypeptide backbone, including amide I and amide III modes, as well as skeletal stretching and deformation vibrations.

Figure 5 presents the Raman spectra of the gelatins. Gelatins exhibited bands at 3292 cm^−1^ attributed to N-H linkages; 2935 cm^−1^ and 1451 cm^−1^ due to C-H bonds; 1666 cm^−1^ corresponding to C=O group; 1247 cm^−1^ due to C-C bonds; and 926 cm^−1^ attributed to C-O-C linkage. Similar to FTIR spectra, gelatins showed changes in the intensity of some Raman bands, with bovine gelatin exhibiting higher intensities at 3292 cm^−1^, 1451 cm^−1^, 1247 cm^−1^, and 926 cm^−1^, followed by fish gelatin and porcine gelatin exhibiting lower intensities.

### 2.4. Thermal Behavior

**Figure 6:**
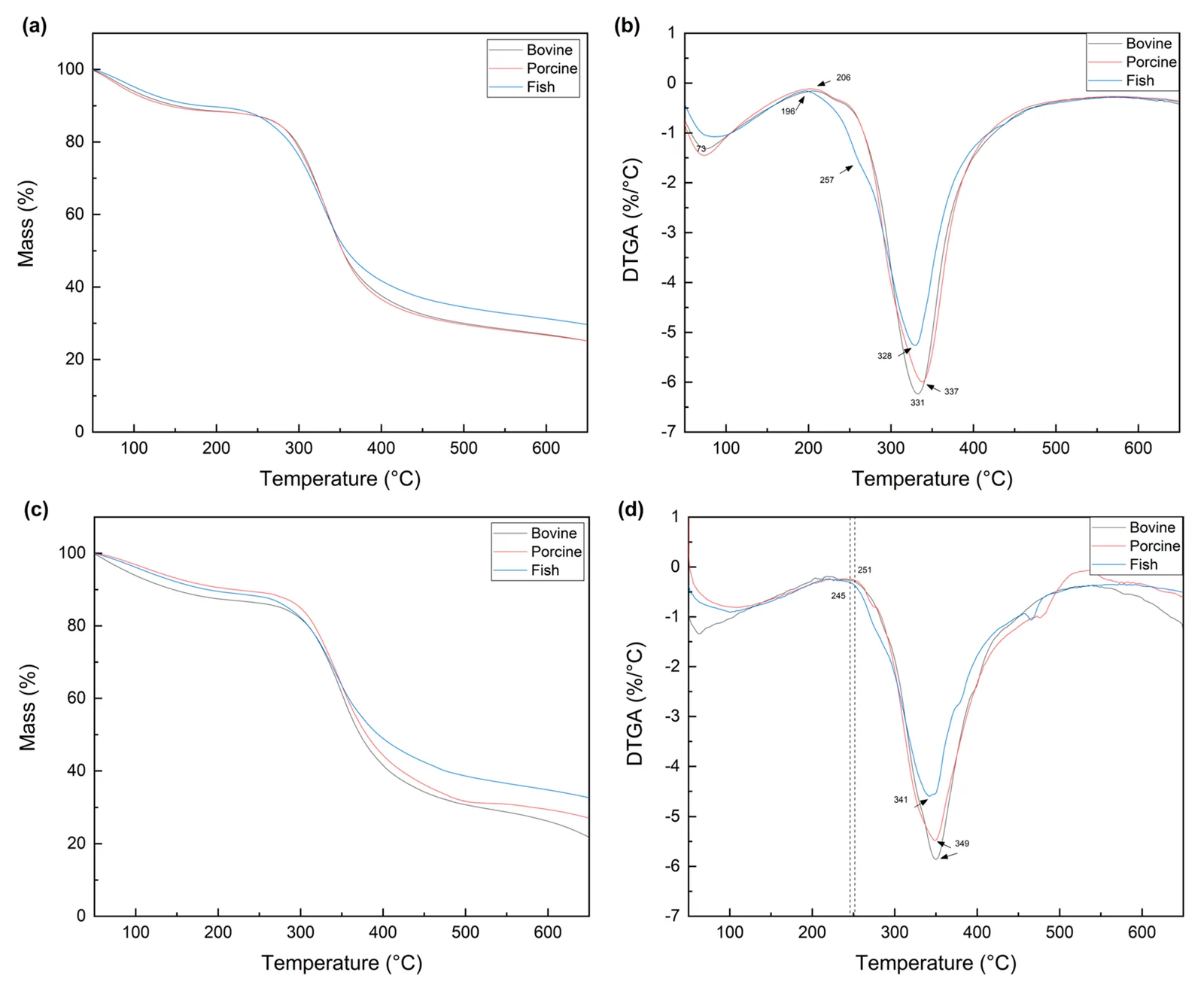
Thermogravimetric analysis (TGA) of gelatin samples. (**a**) TGA and (**b**) DTG curves of pristine, uncrosslinked bovine, porcine, and cold-water fish gelatins. All samples exhibit two main mass-loss events: a first loss associated with the evaporation of bound water and a second major loss corresponding to thermal degradation of the polypeptide backbone. The DTG curves highlight differences in the maximum degradation temperature, with mammalian gelatins degrading at higher temperatures than fish gelatin. (**c**) TGA and (**d**) DTG curves of the corresponding GTA-crosslinked gelatin hydrogels. Crosslinking shifts the onset and maximum degradation temperatures to higher values, indicating enhanced bulk thermal stability relative to the uncrosslinked gelatins.

Figure 6 displays Thermogravimetric analysis (TGA) thermograms of gelatins. All three gelatins exhibited two mass losses, the first one associated with water loss and the second one with the degradation of the gelatin backbone. Mammalian gelatin exhibited a higher onset temperature (206 °C) for the major degradation event, indicating higher thermal stability. In contrast, fish gelatin demonstrated a discernible shift in its derivative thermogram (DTG), with the peak degradation temperature occurring at a lower value (196 °C). This decrease in thermal stability can be attributed to the lower imino acid content, which results in a less rigid polypeptide backbone and a lower density of inter-molecular hydrogen bonds, thereby requiring less energy for pyrolytic decomposition [21,22]. Notably, fish gelatin yielded a higher mineral residue than the mammalian gelatins. Although the amino acid composition of the gelatins was not directly measured in this study, previously published compositional data consistently indicate that cold-water fish gelatin contains a higher proportion of glycine than mammalian gelatins [23]. This higher glycine content may contribute to the increased char yield; glycine-rich regions can undergo condensation and aromatization reactions during pyrolysis, promoting the formation of nitrogen-containing heterocyclic intermediates and a more thermally stable carbonaceous residue. Nevertheless, this interpretation remains tentative without direct compositional analysis, and the contribution of inorganic impurities or ash from the extraction process cannot be excluded. The gelatin hydrogels displayed enhanced thermal stability (as observed in Figure 6c,d), with onset degradation temperatures rising to 251 °C and 245°C and maximum decomposition temperatures reaching 349 °C and 341 °C for the mammalian and fish gelatin hydrogels, respectively. The structural reinforcement afforded by the crosslinked network improves bulk thermal stability.

### 2.5. XRD

**Figure 7:**
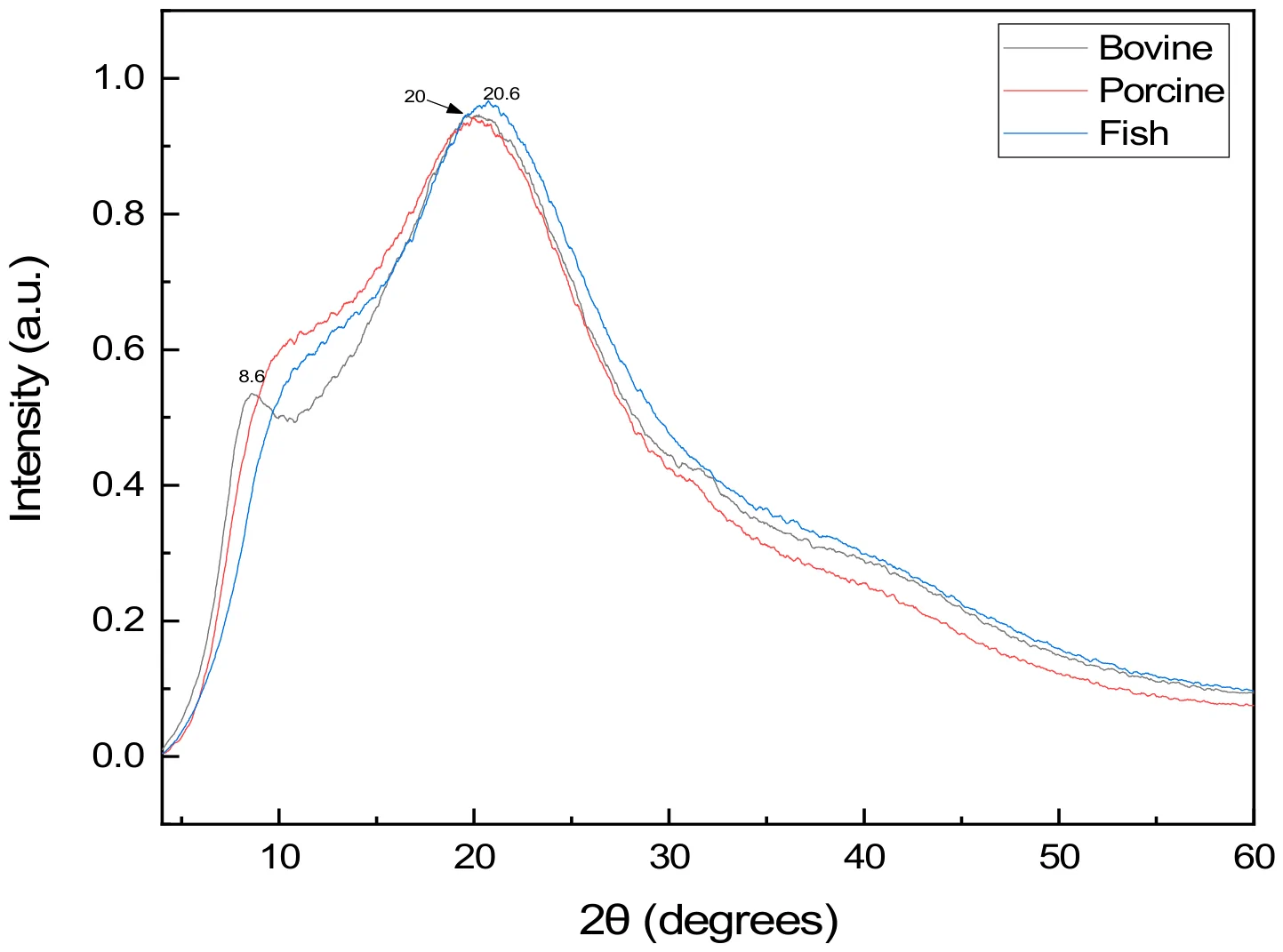
XRD patterns of pristine bovine, porcine, and cold-water fish gelatins. The diffractograms exhibit a characteristic reflection at approximately 8.6° 2θ, associated with the triple-helical structure of gelatin, along with a broad amorphous halo at higher angles corresponding to the disordered polypeptide backbone. The intensity of the triple-helix reflection is highest in bovine gelatin, moderately reduced in porcine gelatin, and appears as a shoulder in fish gelatin, reflecting the progressively lower imino acid content and reduced triple-helical order among the sources. Patterns have been vertically offset for clarity.

XRD patterns of the gelatins are presented in Figure 7. All samples displayed a broad peak characteristic of the amorphous structure, located at approximately 20° 2θ for bovine and porcine gelatins and at a slightly higher angle of 20.6° 2θ for fish gelatin. A second, less intense signal was observed at 8.6° 2θ as a sharp peak in bovine gelatin and as a shoulder near 10° 2θ in the porcine and fish variants. In accordance with previous reports, these two features are assigned to distinct structural elements. The low-angle reflection at ~8° 2θ arises from the lateral packing distance between the collagen-like triple helices and therefore reflects the residual, renatured triple-helical fraction, whereas the broad halo near 20° 2θ originates from the average through-space distance between randomly coiled polypeptide chains in the amorphous domains [24,25,26]. On this basis, the diffractograms indicate two complementary structural differences between the gelatins. First, the triple-helix reflection is sharp and intense in bovine gelatin but reduced to a shoulder in porcine and fish gelatins, indicating a progressively lower content of ordered triple-helical structure; this trend is consistent with the imino acid content of each source, as these residues are required to stabilize the triple helix through interchain hydrogen bonding, and their lower content in cold-water fish gelatin limits helix renaturation upon cooling [6,27,28]. Second, the amorphous halo of fish gelatin is displaced to a higher diffraction angle, corresponding by Bragg’s law to a smaller interchain spacing (from d ≈ 4.4 Å in the mammalian gelatins to d ≈ 4.3 Å in fish gelatin). This tighter average packing of the disordered chains may be attributable to the compositional profile of fish gelatin, namely its lower proportion of the conformationally rigid imino acids and its higher content of glycine, the smallest amino acid, which together allow the random-coil segments to approach one another more closely in the absence of extended helical ordering [6,10,29].

### 2.6. Swelling

**Figure 8:**
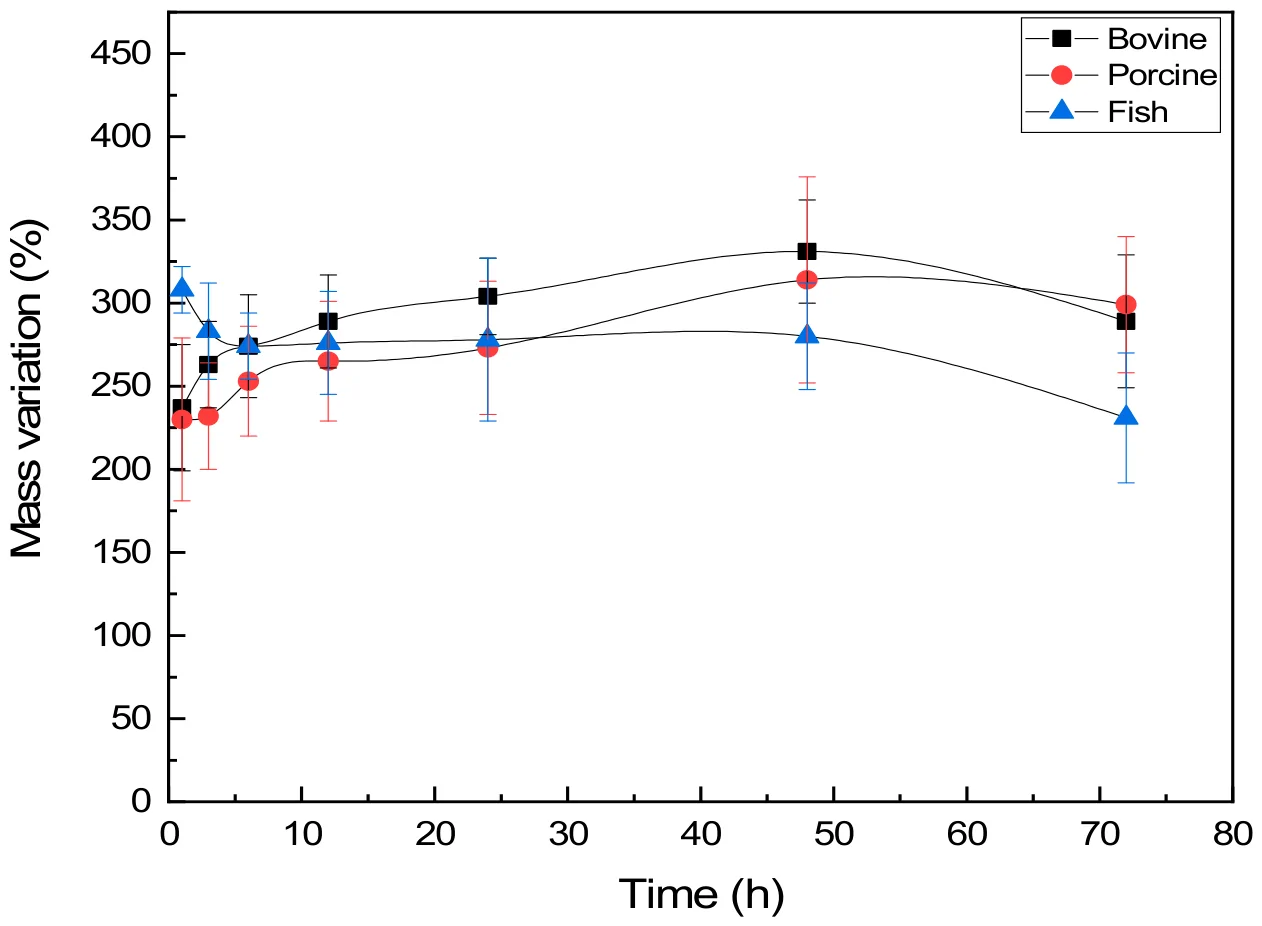
Swelling behavior of GTA-crosslinked gelatin hydrogels immersed in PBS at pH 7.2 and 37 °C. Swelling ratios were determined gravimetrically at predetermined time intervals over 72 h. Mammalian gelatin hydrogels exhibited gradual water uptake and reached swelling equilibrium by 48 h. In contrast, fish gelatin hydrogels displayed rapid initial swelling followed by an early mass loss, attributed to the leaching of soluble, insufficiently crosslinked fractions, and subsequently maintained equilibrium before further mass loss at later time points. Data are presented as mean ± standard deviation (*n* = 5).

The swelling behavior of the hydrogels is presented in Figure 8. The mammalian gelatin hydrogels exhibited gradual water uptake, reaching swelling equilibrium at 48 h. In contrast, the fish gelatin hydrogels displayed a rapid initial swelling to 308% of their initial mass, followed by a mass loss of approximately 33% at 6 h, which can be due to the loss of soluble, uncrosslinked fish gelatin fraction leaching out; thereafter, a swelling equilibrium was sustained up to the 48-h time point. Welz and Ofner [30] demonstrated that self-crosslinked gelatin discs retained only 90% of their original mass after 12 h of immersion in water and phosphate buffer solutions at pH 3 and 6.4, indicating that approximately 10% of the gelatin was lost as soluble material during the initial hydration period. In their study, the mass loss was attributed specifically to the dissolution of gelatin chains not incorporated into the crosslinked network, rather than to hydrolytic chain scission of the network itself. Further support for this mechanism comes from Zhao et al. [31], who studied crosslinked PEG/gelatin hybrid hydrogels and observed that the most significant mass loss (up to 90% at 60 h) occurred progressively through a combination of sol fraction release and subsequent hydrolytic cleavage of the network. Notably, the initial phase of mass loss in their system was dominated by the release of loosely bound or uncrosslinked components before the onset of bulk hydrolytic degradation of the crosslinked matrix.

The additional mass loss observed after 48 h indicates progressive network destabilization, likely resulting from hydrolytic chain scission. This underscores that a sufficient crosslinking density is critical for maintaining long-term structural integrity under physiological conditions. The distinct swelling behavior between the mammalian and fish gelatin hydrogels may be attributed to the higher GTA concentration used to crosslink the fish gelatin. Although the ninhydrin assay indicated a slightly lower crosslinking degree for fish gelatin, the possible formation of GTA oligomers at the higher crosslinker concentration may become physically entangled within the network and restrict chain mobility through non-covalent mechanisms, thereby influencing swelling without increasing the number of covalent amine crosslinks detectable by the ninhydrin assay.

### 2.7. Rheology

**Figure 9:**
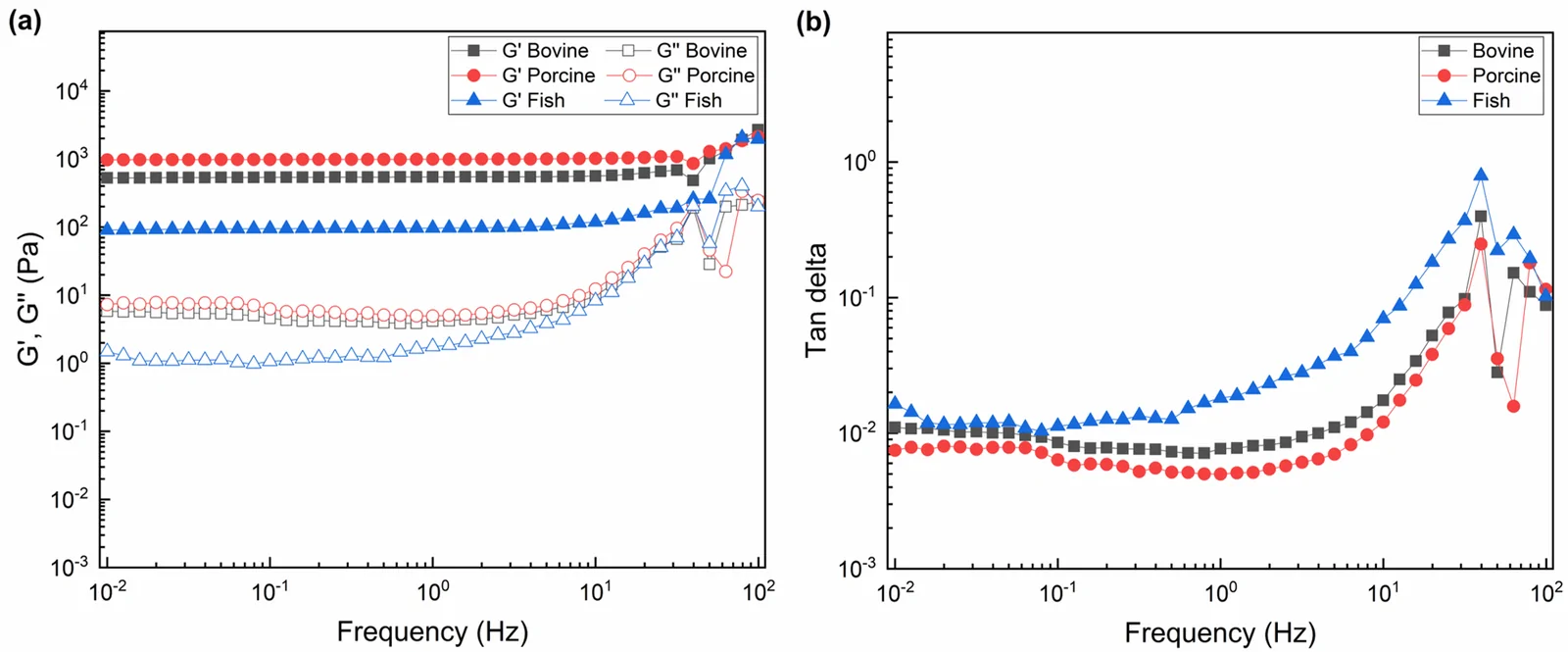
Rheological characterization of GTA-crosslinked gelatin hydrogels. (**a**) Frequency sweep showing storage modulus (G′, closed symbols) and loss modulus (G″, open symbols) as functions of angular frequency for bovine, porcine, and cold-water fish gelatin hydrogels. All hydrogels exhibit G′ values exceeding G″ across the entire frequency range, confirming their solid-like viscoelastic character. (**b**) Loss tangent (tan δ = G″/G′) as a function of frequency. The fish gelatin hydrogels display higher tan δ values than the mammalian formulations, indicating a relatively more dissipative response consistent with their lower mechanical stiffness. Measurements were performed at 25 °C within the linear viscoelastic region.

Figure 9 presents the frequency-dependent viscoelastic behavior of the hydrogels. The rheological response of all hydrogels was frequency-dependent, characterized by a consistent increase in the storage modulus (G′) and a non-monotonic loss modulus (G″) that initially decreased before rising. This profile indicates long-time relaxation of entangled polymer chains: at low frequencies, the extended experimental timescale allows gradual release of topological constraints within the network, maximizing viscous energy dissipation [32,33]. At elevated frequencies, where the deformation rate exceeds the molecular relaxation timescale, the primary mechanism of energy dissipation shifts. Localized segmental motions and intermolecular friction become predominant, accounting for the subsequent rise in G″ [34]. The persistent dominance of G′ over G″ across the entire frequency spectrum unequivocally confirms the material’s solid-like, viscoelastic character [35]. Notably, fish gelatin exhibited a significantly lower G′ than mammalian gelatins, despite being crosslinked with a higher GTA concentration. This mechanical response reflects the combined influence of the intrinsic properties of the gelatin source and the specific crosslinking conditions. Fish gelatin possesses both a lower imino acid content, which limits the formation of load-bearing triple-helical physical junctions, and a lower density of primary amine groups, which restricts the number of covalent crosslinks that can be formed. The higher GTA concentration used for fish gelatin likely promotes glutaraldehyde oligomerization and intramolecular reactions rather than the formation of additional interchain linkages, as evidenced by the slightly lower crosslinking degree measured by the ninhydrin assay. Consequently, although a higher crosslinker concentration was employed, it did not compensate for the reduced availability of reactive sites, resulting in a less mechanically robust network and a lower storage modulus.

### 2.8. Hydrogel Adhesion

**Figure 10:**
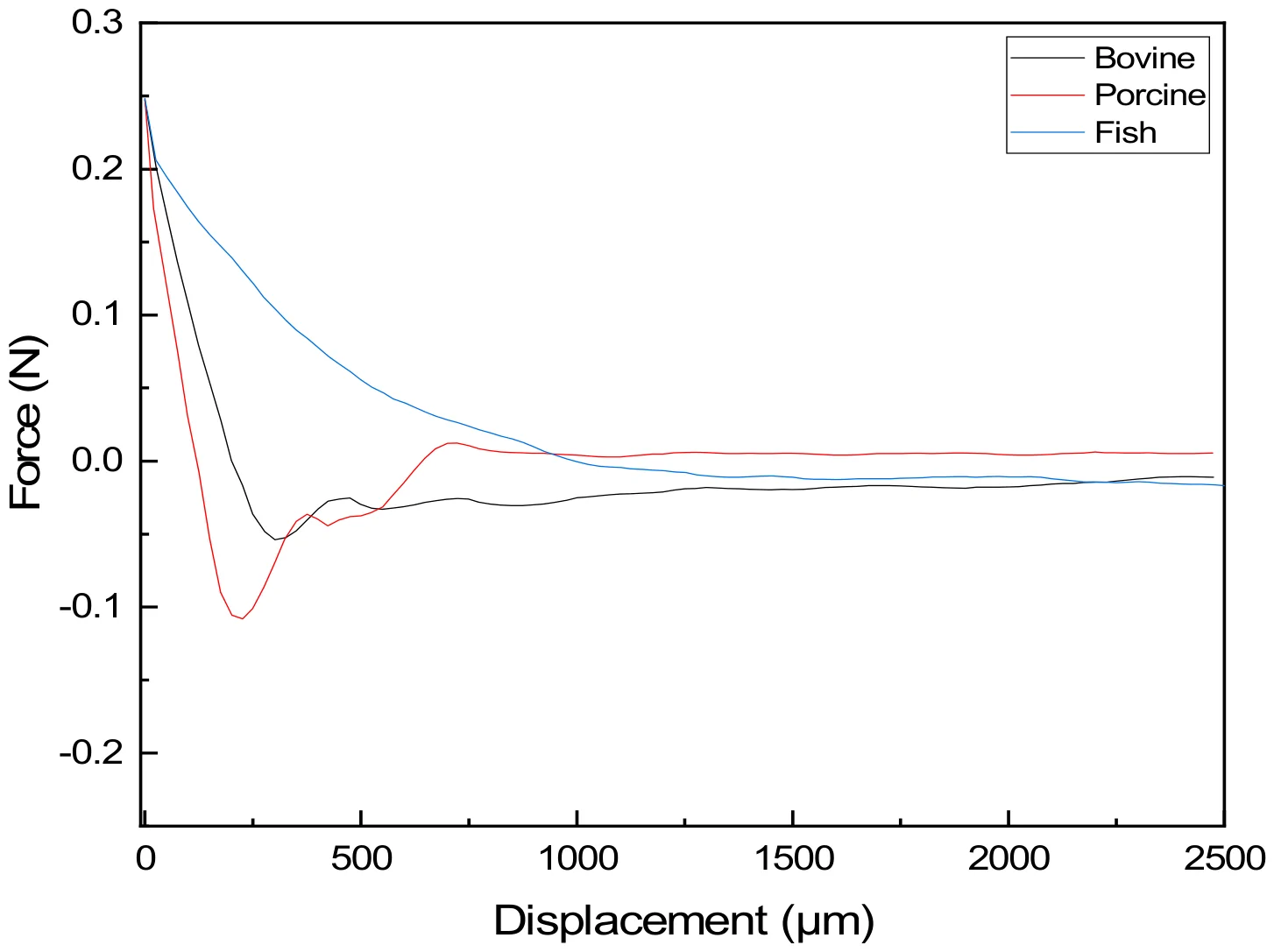
Pull-off adhesion testing of crosslinked gelatin hydrogels. Representative force–displacement curves obtained during detachment of a 25 mm parallel-plate geometry from the hydrogel surface. The hydrogels were first compressed under a normal force of 0.25 Pa, after which the upper plate was retracted at a constant velocity of 100 µm/s. The mammalian gelatin hydrogels exhibited a steeper initial slope and more abrupt detachment, whereas the fish gelatin hydrogels displayed a more gradual force decay and remained adhered to the plate for a longer displacement, indicating a more compliant response and greater surface adhesion.

The results of the pull-off test are presented in Figure 10. The mammalian gelatin hydrogels detached more abruptly from the probe, as reflected by a steeper force–displacement slope (−1.73 ± 0.23 N/mm for porcine and −0.99 ± 0.40 N/mm for bovine, and the force crossed from compression into tension after only 102 ± 17 and 213 ± 108 µm of displacement, respectively), whereas the fish gelatin hydrogels showed a more gradual decay in force, exhibiting a detachment slope of only −0.32 ± 0.03 N/mm and remaining adhered to the plate for an extended period, crossing into tension until 1036 ± 149 µm. This behavior can be ascribed to differences in hydrogel stiffness: the lower proline and hydroxyproline content in fish gelatin limits the formation of stable triple-helical junctions, yielding a softer and more compliant network. Such a network readily wets and conforms intimately to the substrate, causing the interfacial adhesion to surpass the bulk cohesive strength. This suggests that separation occurs predominantly through cohesive (internal) failure rather than interfacial debonding, which dissipates the applied force more gradually. This interpretation is consistent with reports that cold-water fish gelatin exhibits substantially lower gel strength and reduced proline/hydroxyproline content than bovine and porcine gelatins, accounting for its diminished cohesive and thermal stability [36].

### 2.9. Cell Viability

**Figure 11:**
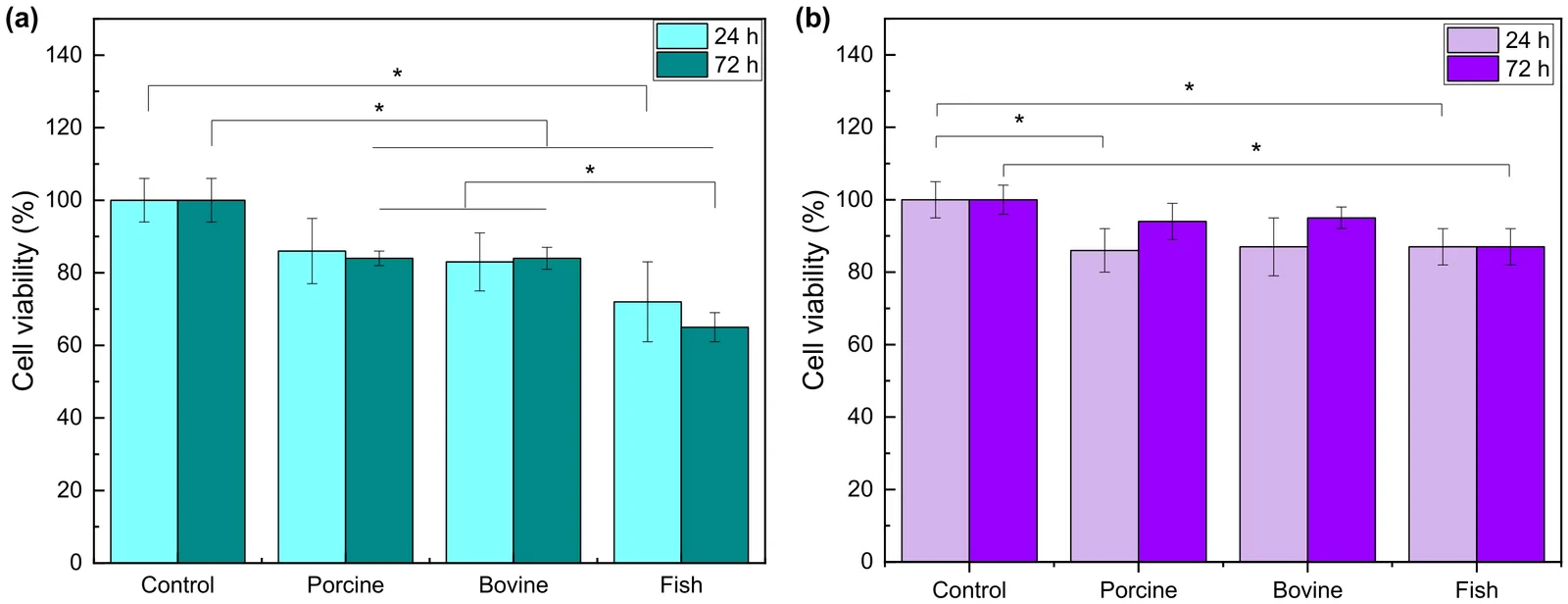
Cell viability of (**a**) osteoblasts and (**b**) fibroblasts cultured on gelatin hydrogels prepared from bovine, porcine, and cold-water fish gelatin. Viability was assessed using a standard metabolic activity assay and is expressed relative to the control condition. Data are presented as mean ± standard deviation. Asterisks (*) indicate statistically significant differences at *p* < 0.05.

The viability of osteoblasts and fibroblasts cultured on the gelatin hydrogels is presented in Figure 11. All gelatin types demonstrated to be non-cytotoxic according to ISO 10993-5, supporting cell viability above 70% of control. Notably, mammalian gelatins promoted significantly higher osteoblast viability than fish gelatin at later time points, although no significant difference was observed at 24 h. In contrast, fibroblast viability remained consistent across all gelatin sources, showing no statistically significant variation.

**Figure 12:**
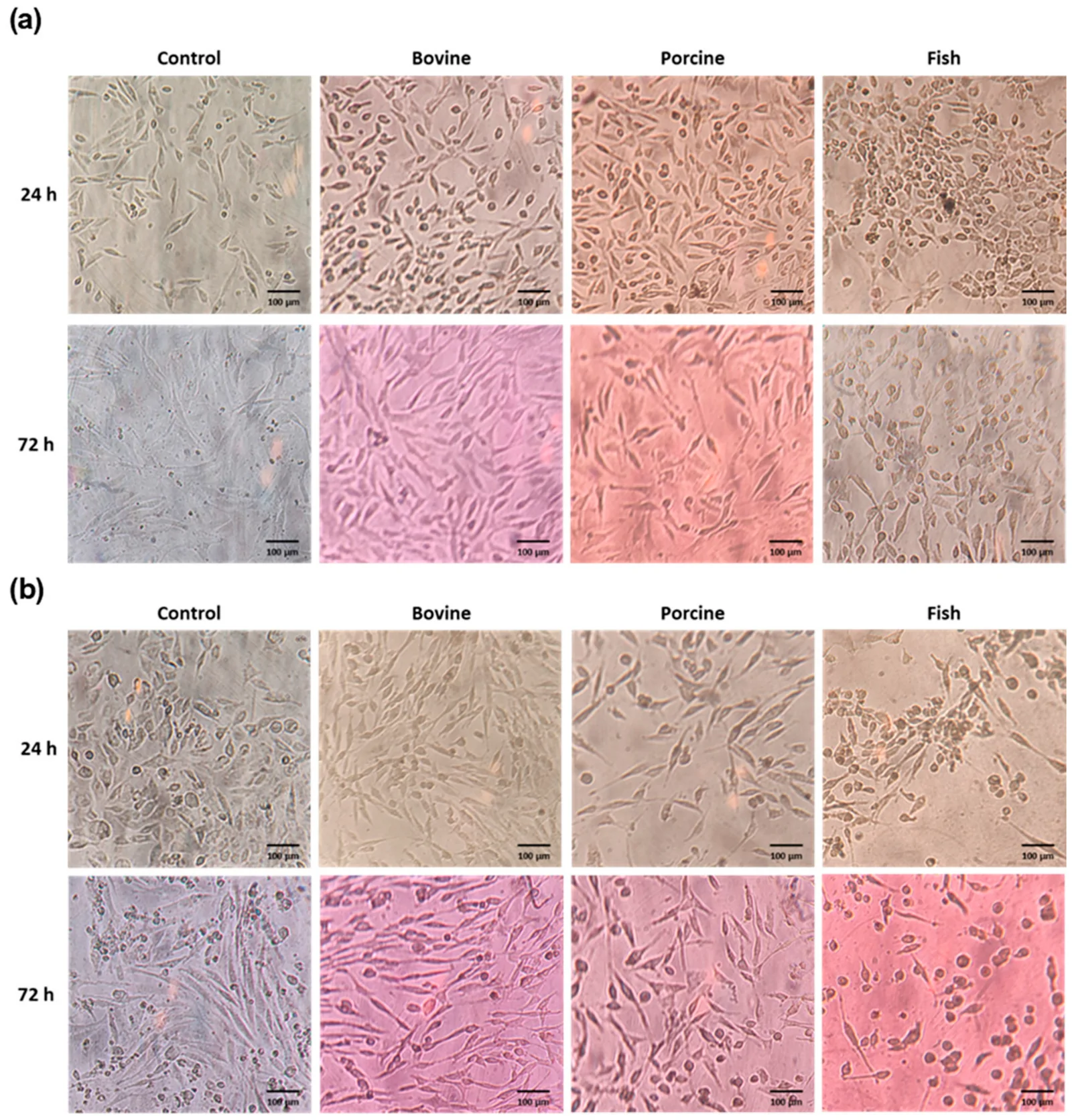
Representative microscopic images of (**a**) osteoblasts and (**b**) fibroblasts cultured on gelatin hydrogels prepared from bovine, porcine, and cold-water fish gelatin at 24 and 72 h. Images illustrate differences in cell attachment, spreading, and morphology among the hydrogel formulations over time.

The morphology of osteoblasts and fibroblasts cultured on the gelatin substrates is shown in Figure 12, respectively. At 24 h, cells on mammalian gelatins predominantly exhibited a fusiform phenotype, similar to the control, though a fraction of cells remained rounded. This initial rounding in osteoblasts was followed by full spreading and a morphological shift to the fusiform shape by 72 h. Quantitative image analysis at 72 h showed that fibroblasts cultured on fish gelatin had a mean cell area of ~86% of that of fibroblasts cultured on mammalian gelatins, whereas in osteoblasts this value decreased to ~75%. These results confirm that both cell types achieved greater spreading on mammalian gelatins than on fish gelatin, indicating poorer adhesion or adaptation.

These morphological differences can be attributed to the lower primary amine content in fish gelatin. Osteoblasts are known to adhere preferentially to positively charged, amine-rich surfaces [37,38,39,40]. Furthermore, crosslinking can reduce the availability of RGD motifs in gelatin, which are crucial for cell adhesion [41,42]. However, the lower content of free primary amine groups in cold-water fish gelatin, in addition to its higher softness, may present cell-adhesive ligands less effectively. Thus, although a higher crosslinker concentration was required to stabilize fish gelatin, the combined effect of lower amine availability and a less robust network is more likely to account for the diminished cell adhesion. Additionally, the potential cytotoxicity of GTA crosslinking, arising from the release of unreacted aldehydes, should be considered; however, it has been reported that thorough rinsing mitigates this risk [43,44,45].

### 2.10. Discussion

The selection of a gelatin source is a fundamental design parameter in biomaterial science, as it dictates the physicochemical properties of a material and, consequently, its biological performance. This study establishes a clear link between the biological source of gelatin and its resulting physicochemical and biological properties. The key differentiating factor is the amino acid profile, which was corroborated by FTIR and Raman analysis. Specifically, the high imino acid (proline and hydroxyproline) content in mammalian gelatins confers greater structural integrity and thermal stability, as evidenced by TGA. XRD analysis further revealed that mammalian gelatins retain a greater proportion of the residual collagen triple-helical structure. This superior structural hierarchy directly enhances their mechanical properties, as confirmed by rheological characterization showing the G’ of fish gelatin to be an order of magnitude lower than that of mammalian gelatins.

These inherent characteristics of fish gelatin present significant challenges for its use as a scaffold in tissue engineering. Its low gelling temperature (~5 °C) necessitates a higher crosslinking density to achieve stability at physiological and room temperatures [6,46]. This requirement has a critical downstream effect, since many crosslinking agents (e.g., glutaraldehyde) target the primary amine groups; the higher crosslinking degree occludes a greater proportion of these polar groups and could mask the RGD motifs essential for cell adhesion [43,44,45]. This phenomenon is exacerbated because fish gelatin intrinsically contains fewer free primary amines (–NH_2_), as quantified by the ninhydrin assay. Collectively, these molecular-level drawbacks translate to an impaired cellular response, manifested as reduced osteoblast viability and a contracted, less-spread morphology in both osteoblasts and fibroblasts. This trend aligns with existing literature. For example, Le Thi et al. [47] reported a higher proliferation of human dermal fibroblasts in porcine gelatin hydrogels compared to fish gelatin counterparts over a seven-day culture period. Similarly, Ma et al. [48] found a slight increase in the viability of MC3T3 pre-osteoblasts cultured on porcine gelatin hydrogels compared to cold-water fish gelatin hydrogels at days 3 and 5. Furthermore, Wang et al. [17] reported significantly greater proliferation of NIH 3T3 fibroblasts on porcine gelatin hydrogels than on fish gelatin in a 2D culture system. Therefore, although cold-water fish gelatin typically exhibits compromised bulk mechanical and thermal properties that limit its use in conventional load-bearing applications, pull-off tests revealed that it adheres to surfaces more effectively than mammalian gelatins. This improved adhesion, rooted in its distinct composition, opens the possibility of employing fish gelatin as a tissue adhesive. Expanding this work to include a broader variety of marine species therefore represents an important direction for future research.

## 3. Conclusions

This study demonstrates that the biological source of gelatin is a primary determinant of its physicochemical, mechanical, and biological performance, governed by a clear structure–function relationship rooted in amino acid composition. TGA and XRD confirmed the higher thermal stability and greater retained triple-helical structure of the mammalian gelatins, explained by their high imino acid content, whereas the imino-acid-poor cold-water fish gelatin formed a less ordered, more compliant network. The ninhydrin assay revealed a lower content of primary amine groups in fish gelatin, corresponding to 70% of that of bovine gelatin. Moreover, the amino acid composition of fish gelatin required twice the crosslinker concentration to achieve dimensional stability (4% *w*/*w* compared to 2% *w*/*w*), which reduces the availability of primary amine groups and could mask critical RGD motifs compromising its bioactivity. This directly translated into a more favorable cellular response for the mammalian gelatins, enhancing osteoblast viability and promoting characteristic adherent morphologies.

Notably, however, the same structural softness that limits the scaffolding performance of fish gelatin conferred a distinct functional advantage in adhesion. In pull-off testing, the mammalian gelatins exhibited high, sharply decaying detachment forces and clean interfacial separation, characteristic of stiff, elastic networks that store and rapidly release strain energy, whereas fish gelatin sustained a lower but more gradual resistive force over a larger displacement, remaining attached to the substrate and adhering readily to handling surfaces. This response indicates that separation of the fish gelatin proceeded predominantly through cohesive rather than interfacial failure, revealing that its interfacial adhesion exceeds its bulk cohesion. The low modulus, intimate surface wetting, and limited elastic recoil of cold-water fish gelatin promote conformal contact and tack on soft, moist substrates. Consequently, the property that constitutes a limitation for load-bearing scaffolds becomes an asset for surface adhesion, positioning fish gelatin as a promising candidate for bio-adhesives.

Taken together, these findings reframe fish gelatin not merely as an inferior substitute for mammalian gelatin but as a material with a complementary property profile. While mammalian gelatins remain preferable where cohesive strength, stiffness, and maximal bioactivity are required, fish gelatin offers a valuable mammalian-free alternative whose softness and surface tack are advantageous for adhesive applications. The rational selection of a gelatin source must therefore be guided by the specific mechanical and biological requirements of the target tissue, balancing bioactivity and cohesive strength against conformability and adhesive performance.

## 4. Materials and Methods

### 4.1. Materials

Type A porcine (G1890, ~300 g Bloom, 50–100 kDa), type B bovine (G9391, ~225 g Bloom) and cold-water fish (G7041, 60 kDa) gelatins were acquired from Sigma-Aldrich (St. Louis, MO, USA). Glutaraldehyde (GTA), ninhydrin, glycine and resazurin sodium salt were also purchased from Sigma-Aldrich (St. Louis, MO, USA). Dulbecco’s Modified Eagle’s Medium (DMEM), fetal bovine serum (FBS), phosphate-buffered saline (PBS), trypsin, penicillin–streptomycin were obtained from Invitrogen (Carlsbad, CA, USA).

### 4.2. Hydrogel Preparation

Gelatin hydrogels were prepared by dissolving gelatin (10% *w*/*v*) in sterile distilled water at 40 °C under constant magnetic stirring. After complete dissolution, the solutions were crosslinked with GTA. Bovine and porcine gelatins were crosslinked with 2% (*w*/*w*) GTA, whereas fish gelatin required 4% (*w*/*w*) GTA to yield a hydrogel stable at 37 °C. The reaction mixtures were immediately cast into Petri dishes and left to gel for 24 h at 25 °C. To ensure biocompatibility, the crosslinked hydrogels were immersed in 0.5 M glycine solution for 24 h to quench residual aldehyde groups, followed by three sequential 2 h washes in PBS at physiological temperature (37 °C) to remove any leachable compounds.

### 4.3. Physico-Chemical Characterizations

#### 4.3.1. FTIR

Fourier Transform Infrared (FTIR) spectra of gelatins were obtained in the range of 4000 cm^−1^ to 650 cm^−1^, a resolution of 4 cm^−1^ and an average of 200 scans, with a Nicolet 8700 spectrometer from ThermoFisher (Madison, WI, USA) equipped with a ZnSe attenuated total reflectance (ATR) accessory. FTIR spectra were normalized by dividing each spectrum by its maximum absorbance value. Relative band intensities were subsequently compared by calculating the ratio of each target band to the maximum intensity of the respective spectrum.

#### 4.3.2. Raman Spectroscopy

Raman spectroscopic analysis was performed using a DXR Raman microscope from a ThermoFisher system equipped with a 633 nm excitation source, employing an exposure time of 10,000 ms at full laser power (100%). Spectral acquisition covered the wavenumber range of 450–3300 cm^−1^. Raman spectra were normalized by dividing each spectrum by its maximum absorbance value.

#### 4.3.3. Ninhydrin Assay

Ninhydrin assay was performed to evaluate the proportion of free primary amine groups (-NH_2_) in the gelatins. Briefly, 1 mL of a ninhydrin solution (1.5% in ethanol) was added to 5 mg of each sample and heated at 80 °C for 20 min. After this, samples were allowed to cool down for 15 min, and the absorbance of the solution at 570 nm was evaluated in a Cytation 3 plate reader from BioTek (Winooski, VT, USA). The results are reported as normalized values relative to the mean absorbance of bovine gelatin (0.6593 ± 0.0264). Five replicates were analyzed per sample.

Crosslinking degree was estimated through the reduction in free amine groups of crosslinked hydrogels compared with the non-crosslinked sample. Crosslinking degree was calculated with the following equation:

(1) $$Crosslinking\;degree\;\left(\%\right)=\left(1-\frac{A}{A_{o}}\right)\times 100,$$

where *A* is the absorbance of the crosslinked sample and *A_o_* is the absorbance of the non-crosslinked sample.

#### 4.3.4. TGA

TGA of the gelatins was performed with Perkin-Elmer TGA 8000 equipment (Waltham, MA, USA), using a heating rate of 10 °C/min from 40 to 650 °C under nitrogen flow.

#### 4.3.5. XRD

XRD was performed using D2 PHASER equipment from Bruker (Billerica, MA, USA). Samples were analyzed in 2θ mode from 4° to 70° with a 1 mm rack, 0.5 s of exposition and an increment of 0.01°. A smoothing was applied to the spectra using the Savitzky–Golay method with 5 points in the Origin (2018) software.

#### 4.3.6. Swelling

Dried gelatin hydrogels were immersed in PBS and allowed to swell at 25 °C for 72 h. The hydrogels were weighed after 3, 6, 12, 24, 48 and 72 h; prior to each measurement, residual surface water was removed by blotting with filter paper. All experiments were performed using five replicates. The swelling ratio was determined according to the following equation:

(2) $$Swelling\;\left(\%\right)=\;\frac{W-W_{0}}{W_{0}}\;\times \;100,$$

where *W* is the swollen weight and *W_0_* is dried weight at the start of the experiment.

#### 4.3.7. Rheological and Pull-Off Test

The viscoelastic properties of the hydrogels were characterized by small-amplitude oscillatory shear rheometry using a rotational rheometer with a 25 mm parallel plate geometry and a 1000 μm gap. Frequency sweep tests were performed within the linear viscoelastic region (LVR) at 1% strain (this was confirmed by a strain–sweep assay, as shown in Figure S1) over an angular frequency range of 0.01 to 100 Hz at 25 ± 0.2 °C.

Adhesion of the hydrogels was evaluated by means of a pull-off (tack) test using a 25 mm parallel-plate geometry. The hydrogels were initially compressed under a normal force of 0.25 N, after which the upper plate was retracted at a velocity of 100 µm/s.

### 4.4. Cell Culture

Human gingival fibroblasts (obtained from Dr. Hideyo Noguchi Regional Research Center, Yucatán, México, CIE-06–2017) and rat osteoblasts (obtained from Instituto Nacional de Ciencias Médicas y Nutrición “Salvador Zubirán”, Mexico City, CINVA-BRE-265) were expanded in DMEM supplemented with 10% FBS, 1% penicillin–streptomycin, and 50 µg/mL of ascorbic acid for osteoblast medium, at 37 °C and 5% CO_2_. When cells reached 80% confluence, they were detached using a 0.25% trypsin-EDTA solution and viable cells were counted using a mixture of trypan blue and cell suspension (1:1) in a hemocytometer (Neubauer cell chamber).

### 4.5. Cell Viability

Gelatin hydrogels were UV-sterilized for 30 min, placed in a 96-well plate, and rinsed twice with PBS. Osteoblasts and fibroblasts were seeded on the hydrogels at a density of 4 × 10^3^ cells and incubated at 37 °C and 5% CO_2_ with 100 µL of culture medium. Morphology of the cells was examined after 24 h and 72 h using a Labomed TCM 400 microscope (Los Angeles, CA, USA). Average cell area was quantified by segmenting microscopy images using Cellpose version 4.0.6, with flow threshold of 2.5 and cellprob threshold of 0.0; the total detected cell area was then divided by the number of cells. Metabolic activity was assessed by adding 20 µL of CellTiterBlue^®^ to each well, followed by 4 h incubation. After incubation, 50 µL of medium were transferred from each test well to fresh wells in a clean plate to minimize optical interference from the hydrogels. Cells cultured on tissue culture polystyrene were used as positive controls, and five replicates were prepared for each sample. Absorbance measurements were then performed at 570 nm using a Cytation 3 (BioTek) plate reader. Cell viability was determined using the following equation:

(3) $$Cell\;viability\;\left(\%\right)=\;\frac{A-A_{o}}{A_{c}-A_{o}}\;\times \;100,$$

where *A* represents the absorbance of the sample, *A_o_* represents the absorbance of the test well without cells, and A_c_ is the absorbance of the control cells.

### 4.6. Statistical Analysis

Quantitative data are reported as mean ± standard deviation (n ≥ 5). Intergroup differences were assessed via one-way ANOVA with Tukey’s post hoc test, where *p* < 0.05 denoted statistical significance.

## Data Availability

The raw data supporting the conclusions of this article will be made available by the authors on request.

## Supplementary Materials

The following supporting information can be downloaded at https://www.mdpi.com/article/10.3390/gels12090789/s1, Figure S1: Strain-sweep assay of GTA-crosslinked hydrogels.

## Abbreviations

The following abbreviations are used in this manuscript:

GelMA	Gelatin methacryloyl
GTA	Glutaraldehyde
FTIR	Fourier transform infrared
TGA	Thermogravimetric analysis
DTG	Derivative thermogram
XRD	X-ray diffraction
G’	Storage modulus
G’’	Loss modulus
RGD	Arginine–glycine–aspartic acid
DMEM	Dulbecco’s Modified Eagle’s Medium
FBS	Fetal bovine serum
PBS	Phosphate-buffered saline
EDTA	Ethylenediaminetetraacetic acid
ANOVA	Analysis of variance
