# Supplementary material for: Effect of Gelatin Source on Hydrogel Properties and Cell Viability: A Comparison of Bovine, Porcine, and Cold-Water Fish Gelatin Hydrogels
Source: Gels. 2026 Sep 1;12(9):789. doi: 10.3390/gels12090789 (PMC13606164; doi:10.3390/gels12090789)
Supplement: Supplementary file 1 [file gels-12-00789-s001.zip › gels-4495835-supplementary.pdf]

Supplementary Material

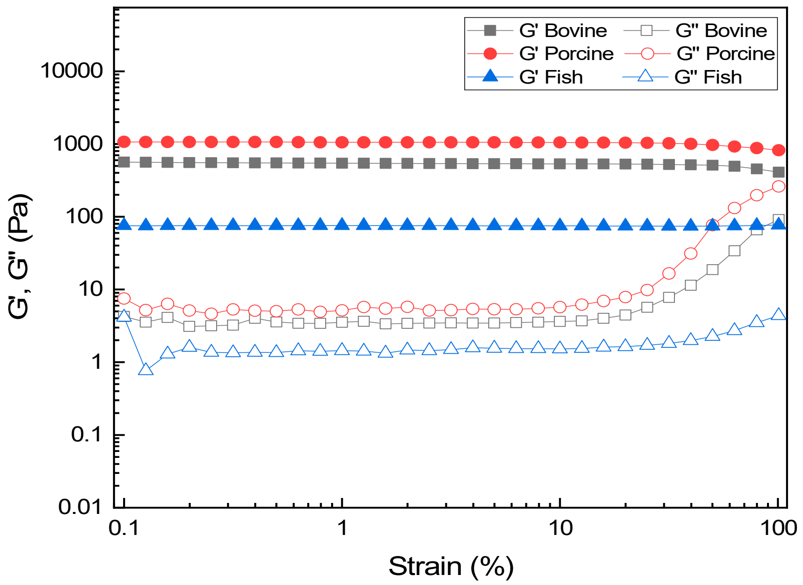

Figure S1. Strain-sweep assay of GTA-crosslinked hydrogels.
